# Asymmetries in Responses to Attitude Statements: The Example of “Zero-Sum” Beliefs

**DOI:** 10.3389/fpsyg.2016.00984

**Published:** 2016-06-29

**Authors:** Michael Smithson, Yiyun Shou

**Affiliations:** Research School of Psychology, The Australian National UniversityCanberra, ACT, Australia

**Keywords:** attitudes, beliefs, zero-sum, measurement, attitude bias

## Abstract

While much has been written about the consequences of zero-sum (or fixed-pie) beliefs, their measurement has received almost no systematic attention. No researchers, to our awareness, have examined the question of whether the endorsement of a zero-sum-like proposition depends on how the proposition is formed. This paper focuses on this issue, which may also apply to the measurement of other attitudes. Zero-sum statements have a form such as “The more of resource X for consumer A, the less of resource Y for consumer B.” X and Y may be the same resource (such as time), but they can be different (e.g., “The more people commute by bicycle, the less revenue for the city from car parking payments”). These statements have four permutations, and a strict zero-sum believer should regard these four statements as equally valid and therefore should endorse them equally. We find, however, that three asymmetric patterns routinely occur in people's endorsement levels, i.e., clear framing effects, whereby endorsement of one permutation substantially differs from endorsement of another. The patterns seem to arise from beliefs about asymmetric resource flows and power relations between rival consumers. We report three studies, with adult samples representative of populations in two Western and two non-Western cultures, demonstrating that most of the asymmetric belief patterns are consistent across these samples. We conclude with a discussion of the implications of this kind of “order-effect” for attitude measurement.

“There's nothing surer, the rich get richer and the poor get—children”

Raymond B. Egan and Gus Kahn, “Ain't We Got Fun.”

## Asymmetries in zero-sum beliefs

A zero-sum belief is one in which the total amount of available resources is considered to be fixed, and gains to one party are matched by others' losses. Some resources are genuinely zero-sum. As any child knows, if a larger piece of Mother's freshly baked cake is given to a sibling, then less is available for him or her. Other resources, such as wealth, friendship, and success, are not necessarily zero-sum but are perceived so by some people under at least some conditions.

Zero-sum thinking is topical, has been given book-length popular treatments (e.g., Wright, 2000), and has featured in prominent forums for current affairs. If a situation is perceived as zero-sum, those involved are likely to act in competition, and positive outcomes for both parties are unlikely to result from negotiation (Gries, 2005). A Forbes online columnist (Kalgaard, 2006) called it the “worst disease” and laid its genesis at the feet of politicians and their advisors who “occupy a zero-sum world…in which one person's gain is another's loss.” A key dividing-point between illiberal and liberal politics is whether wealth is zero-sum. Zero-sum perceptions have also been claimed to contribute to inter-group prejudice (Bobo and Hutchings, 1996) and public resistance to immigration (Esses et al., 2001).

While much has been written about the consequences of zero-sum beliefs, the causes of zero-sum beliefs and psychological influences on them have received little attention, and likewise issues involving the measurement of such beliefs. In one of the few studies investigating causes of zero-sum beliefs, Meegan (2010) speculates that zero-sum bias is linked to competitiveness, and may have arisen as a form of cognitive adaptation. A tendency for zero-sum thinking may well be predicted by specific attitudes, cognitive styles or even personality traits, but this possibility has not been systematically explored. In one of the few relevant studies, Esses et al. (2001) report that individuals high in Social Dominance Orientation are more likely to show zero-sum tendencies when expressing attitudes about immigration.

Rozycka-Tran et al. (2015) recently report a 37-nation scale-validation study of a 12-item “belief in zero-sum game” scale. However, no researchers, to our awareness, have examined the question of whether the degree to which people think a specific proposition is zero-sum depends on how the proposition is formed. This paper focuses on this question. Zero-sum candidate statements have the form “The more of resource X for consumer A, the less of resource Y for consumer B.” X and Y may be either the same resource (such as time) or different resources (e.g., “The more people commute by bicycle, the less revenue for the city from car parking payments”). These statements have four permutations when resource X is linked with consumer A and Y with B:

1. “The more X for A, the less Y for B”
2. “The less X for A, the more Y for B”
3. “The more Y for B, the less X for A”
4. “The less Y for B, the more X for A”

A strict zero-sum believer should regard these four statements as equally valid and therefore should endorse them equally. For instance, if X and Y both are time, and A is being awake and B is sleeping, then we should regard all four statements as equally valid (e.g., “The more time for sleeping, the less time for being awake” = “The more time for being awake, the less time for sleeping”). We wish to make it clear that whether a statement is zero-sum often is very much in the eye of the beholder. The subject of our investigation is *subjective beliefs* about statements that may or may not be zero-sum, rather than perceptions of only those statements that are “objectively” zero-sum. The major claim in this paper is that such statements often are endorsed unequally in systematic ways even when they arguably are zero-sum, depending on how the four elements (X, A, Y, and B) are permuted and how people construe them.

Our claim is important for four reasons. First, it alerts researchers to the fact that measuring zero-sum beliefs involves some subtleties, because rearranging the elements of zero-sum propositions can substantially affect people's degrees of self-reported belief in them. Second, it suggests that some situations may be framed as zero-sum by describing them in particular ways, but as not zero-sum if described in other ways. A judicious choice of description therefore could head off zero-sum thinking before it can affect a negotiation or relationship. Third, this claim extends the topic of research on zero-sum beliefs to include “partial” or “conditional” zero-sum beliefs (also see Smithson et al., 2015). We will return to these points at the end of the paper.

Fourth (and not least), this type of “order-effect” may crop up in the measurement of other attitudes, and this paper presents effective methods for investigating it. Order-effects are commonplace in research on attitude measurement, decision making, and consumer product evaluation and choice. For example, a simple “primacy effect” is well-known, such that the first alternative in a sequence of alternatives tends to be preferred over the others (e.g., Mantonakis et al., 2009). Likewise, people evaluate joint outcomes as better if they occur in an improving sequence (e.g., worse-better) than if they occur in a deteriorating sequence (better-worse; e.g., Hsee and Abelson, 1991; Loewenstein and Prelec, 1993).

The type of order-effect we anticipate is partly due to the fact that many zero-sum statements have the same form as modus-ponens propositions. People may reasonably endorse a modus-ponens proposition less strongly when the antecedent and consequent are reversed (e.g., “If it is a seagull, then it is a bird” vs. “If it is a bird, then it is a seagull”). Many attitude measurement scales contain modus-ponens-like propositions, and permuting the order of their components could influence endorsement. Consider, for instance, “Cheating is not justified because it is unfair to others” vs. “Cheating is unfair to others because it is not justified” (the former is an item from Levenson et al., 1995), or “If I feel competent, I am willing to take responsibility for making decisions” vs. “If I am willing to take responsibility for making decisions, I feel competent” (the former is an item from Raskin and Terry, 1988).

We now turn to the task of building a case for our claim. Consider the propositions “If your best friend increases the attention they pay to someone else, they pay less attention to you” vs. “If your best friend decreases the attention they pay to someone else, they pay more attention to you.” The former may make sense because there is a sense that the friend's attention is being tied up or committed to another person, so that is attention no longer available for bestowing on you. In the latter, the connection seems weaker. Investing less attention to someone else does free it up, but it may be spent on someone (or something) other than you. The intuition here is that if one consumer (e.g., someone else) increases his/her possession of a resource (your friend's attention), another consumer (you) must end up with less of it than could otherwise have been the case; whereas if a consumer decreases his/her share of the resource, it does not necessarily accrue to another particular consumer. We shall call this intuition the “asymmetric resource flow” (ARF) heuristic.

We use “heuristic” here similarly to its use in the decision-making literature, i.e., to mean a common-sense intuition about how the world works in the form of a rule-like proposition that can be used to form judgments or make decisions. Although heuristics in this sense sometimes are associated with biases, we are not claiming that the ARF or any other heuristic examined in this paper involves or results in bias.

A second heuristic arises when people distinguish one consumer as more potent or powerful than the other, thus positioning the first consumer as a cause and the other as an effect. In other words, they may view zero-sum propositions as causal propositions and apply intuitive causal reasoning to them. Consider the propositions “Devoting more time to work takes time away from personal relationships” vs. “Devoting more time to personal relationships takes time away from work.” You may view time spent at work as controlled by external factors (such as your supervisor), while time spent with personal relationships as a matter of free choice. Increased demands for you to work more hours will subtract from time available for personal relationships, but increasing the time you put into relationships will not affect your required working hours. These considerations would lead you to agree more strongly with the first than with the second proposition. We shall call this the “asymmetric consumer potency” (ACP) heuristic.

From here on, we will need some notation to represent the permutations of the elements in our propositions, thereby enabling a short-hand representation of differential endorsements of these permutations. To simplify, we will restrict discussion to cases where there is just one resource (i.e., where X = Y), or where each resource is tied to only one consumer (e.g., A always is linked with X and B always is linked with Y). Our four propositions are displayed in Table 1 with corresponding two-letter notations denoting degree of endorsement. The upper-case letter represents “more X” and the lower-case represents “less X.” For example, Ab represents the strength of endorsement of the proposition that “The more X for A, the less X for B.” Although we do not treat such cases in this paper, if resources X and Y were free to be permuted independently of consumers A and B then we would need to extend this notation to index them as well, so that we could distinguish between permutations such as “The more X for A, the less Y for B” (denoted by AXby) from “The more Y for A, the less X for B” (denoted by AYbx).

**Table 1:** **Notation for strength of endorsement of zero-sum propositions**.

**Statement**	**Notation**
“The more X for A, the less X for B”	Ab
“The less X for A, the more X for B”	aB
“The more X for B, the less X for A”	Ba
“The less X for B, the more X for A”	bA

Thus, a test of whether the ARF heuristic holds is whether Ab > aB and Ba > bA, because the order of A and B is constant in each comparison. Likewise, a test of whether the ACP heuristic holds, with A more potent than B, is if Ab > Ba and aB > bA, and the converse if B is more potent than A.

We report three studies. The first study is a “proof of concept” investigation, testing whether the ACP and/or ARF patterns are readily detectable in a variety of zero-sum statements and their permutations. A straightforward method of ascertaining the existence of such differential endorsement patterns is through two-alternative forced choice experiments, and this is the approach employed in Study 1. Studies 2 and 3 utilize conventional ordinal Likert scales measuring the degree to which participants agree or disagree with statements. The use of rating-scales enables a more nuanced analysis of any deviations from equality of endorsements. To ensure that findings are not an artifact of the number of points on the rating-scale, a 5-point Likert format is employed in Study 2 and a 7-point format in Study 3.

## Study 1

### Method

This study was conducted as an online experiment administered through a Qualtrics™ panel, subsequent to approval by The Australian National University Human Research Ethics Committee. The Qualtrics fee charged to researchers per participant was $7.50USD. There were 508 completed surveys by American participants (at least 18 years old), with a mean age of 39.9 years (SD = 15.0), 50% of whom were female. The median and interquartile deviation for the time participants spent on the survey were 15.6 and 9.8 min, respectively. Participant data were excluded from the analyses if the participant spent less than 5 min on the survey, “flat-lined” responses, or did not answer “paying attention” questions correctly. The 5-min criterion was established on the basis of pilot-testing, wherein we established that even someone familiar with the questionnaire would take at least 5 min to complete it. “Flat-lining” refers to giving the same response on Likert scales throughout an entire battery of items. The usable cases included 205 females and 190 males (total *N* = 395).

Table 2 displays the experimental design. The ACP column contains the tests of the ACP heuristic's components, and the ARF column contains the tests of the asymmetric resource commitment heuristic's components. Both experimental factors are between-subjects factors. The cells in the table describe 2-alternative forced choice that participants were asked to make, i.e., choosing which of the two permutations they agree with the most strongly.

**Table 2:** **Study 1 experimental design and logistic regression dummy variables**.

	**ACP**	**ARF**
Ab comparisons	Ab vs. Ba (*x*_1_)	aB vs. Ab
bA comparisons	aB vs. bA (*x*_2_)	bA vs. Ba (*x*_3_)

Examples of the zero-sum-like statements employed in this study are shown in Table 3. The six statements were selected from results of a pilot study (details of which available from the first author) in which participants were asked to choose between pairs of alternative permutations of various statements. Statements were selected on the basis of how clear they were to participants and an absence of unanimous agreement or unanimous disagreement with them.

**Table 3:** **Study 1 zero-sum propositions**.

**Item**	**Label**	**X/Y**	**Example (Ab)**
S1	Work-Personal	Time	Devoting more *time* to **work** (A) takes time away from **personal relationships** (b).
S2	Friends-Family	Time	Spending more *time* with **friends** (A) takes time away from **family** (b).
S3	Investment	Money	Investing more *money* in one **venture** (A) means there is less for **the others** (b).
S4	Best Friend	Attention	If my best friend increases the *attention* they pay to **me** (A), they pay less *attention* to **someone else** (b).
S5	Immigration-Jobs	Immigration/Jobs	If the rate of **immigration** (A) is increased there will be fewer **jobs** (b) to go around.
S6	Rich-Poor	Wealth	When the **rich** (A) get richer the **poor** (b) get poorer.

Participants were randomly assigned to one of the four conditions. After an introductory screen asking for informed consent, the participants were asked on six occasions to choose which of a pair of statements they agreed with the most. Each pair consisted of two appropriate permutations of the *j*^th^ statement (for *j* = 1, …, 6) from Table 3. Participants were then asked to provide their age and gender, after which they were thanked and debriefed via a closing information screen.

### Results and study 1 discussion

Given that the dependent variable is binary and that the parameters being estimated are probabilities of choosing one alternative vs. the other, a normal-distribution linear regression model is inappropriate. Instead, the six statement comparison sets were initially analyzed with binary logistic regressions. The purpose of these was to test for unequal splits among the four permutations in each set. An absence of unequal splits would indicate that either there were no preferences for one permutation over another or the rather unlikely outcome that the ACP and ARF heuristics both applied to the same degree. The logistic regressions had the following form:

(1) $$\begin{array}{l} y_{i}^{\prime }=\beta_{0}\;+\;\beta_{1}x_{1i}\;+\;\beta_{2}x_{2i}+\;\beta_{3}x_{3i}, \end{array}$$

where *y*′ denotes the logit of the expected probability of choosing the left-most permutation in the pairs in Table 2 (e.g., choosing Ab rather than Ba in the first cell in Table 2), and *x*_1_, *x*_2_, and *x*_3_ are {0,1} dummy variables that take a value of 1 for the comparison they are listed with in Table 2 (e.g., *x*_1_ = 1 when the choice is between Ab vs. Ba, and otherwise *x*_1_ = 0). For statement S1, the logistic model yielded $y_{i}^{\prime }\;=\;-0.294+2.206x_{1i}\;+\;1.744x_{2i}\;+\;0.048x_{3i},$ and the likelihood ratio-test comparing this model against its null, $y_{i}^{\prime }=\beta_{0}$, counterpart resulted in $\chi_{\left(3\right)}^{2}$ = 75.752 (*p* < 0.001). Five of the comparison sets' logistic regressions yielded significant effects from the experimental manipulations [in comparisons against their null models, all $\chi_{\left(3\right)}^{2}$ > 21.0, *p* < 0.001], the exception being S3 [“Investing more money…,” $\chi_{\left(3\right)}^{2}$ = 3.6, *p* = 0.312].

Table 4 displays the percentages and confidence intervals for the relevant choices involving the six statements. These percentages are linked to the logistic regressions in the usual way. For instance, the percentages in the first row of Table 4 are derived as follows. The model for S1 described above yields exp(−0.294 + 2.206)/(1 + exp(−0.294 + 2.206)) = 0.871 for the probability of choosing Ab over Ba (thus, 87.1%), and 1/(1 + exp(−0.294)) = 0.573 for the probability of choosing Ab over aB (57.3%).

**Table 4:** **Choice percentages and 95% Confidence Intervals**.

	**ACP**	**ARF**
**S1: WORK-PERSONAL**		
Ab	Ab > Ba: 87.1% [79.2%, 92.3%]	Ab > aB: 57.3% [47.3%, 66.7%]
bA	aB > bA: 81.0% [72.2%, 87.5%]	Ba > bA: 56.1% [46.3%, 65.5%]
**S2: FRIENDS-FAMILY**		
Ab	Ab > Ba: 79.2% [70.3%, 86.0%]	Ab > aB: 38.8% [29.7%, 48.7%]
bA	aB > bA: 86.0% [77.9%, 91.5%]	Ba > bA: 74.0% [64.4%, 81.7%]
**S3: INVESTMENT**		
Ab	Ab > Ba: 51.0% [41.3%, 60.7%]	Ab > aB: 52.5% [42.8%, 61.9%]
bA	aB > bA: 61.5% [51.5%, 70.6%]	Ba > bA: 49.0% [39.4%, 58.7%]
**S4: BEST FRIEND**		
Ab	Ab > Ba: 54.1% [44.2%, 63.6%]	Ab > aB: 64.0% [54.2%, 72.7%]
bA	aB > bA: 64.6% [54.6%, 73.4%]	Ba > bA: 61.4% [51.6%, 70.3%]
**S5: IMMIGRATION-JOBS**		
Ab	Ab > Ba: 74.5% [65.0%, 82.1%]	Ab > aB: 50.5% [40.9%, 60.1%]
bA	aB > bA: 78.1% [68.9%, 85.2%]	Ba > bA: 56.0% [46.2%, 65.3%]
**S6: RICH-POOR**		
Ab	Ab > Ba: 83.2% [74.7%, 89.2%]	Ab > aB: 80.6% [71.7%, 87.2%]
bA	aB > bA: 35.0% [26.4%, 44.7%]	Ba > bA: 21.9% [14.8%, 31.1%]

Components of the ACP pattern are found in 9 out of 12 comparisons, mostly with substantial majorities. The single counter-example is the aB vs. bA comparison in S6. A complete ACP test requires both Ab > Ba and aB > bA, and we can see in Table 4 that this test is passed by S1, S2, and S5. Components of the ARF pattern are found in 3 out of 12 comparisons, with a complete ARF pattern in S4. There are two counter-examples, the Ab vs. aB comparison in S2 and the Ba vs. bA comparison in S6. We therefore have evidence suggesting that both the ACP and ARF patterns can be manifested in zero-sum-like statements. In contrast, S3 is the only statement that fitted a strict zero-sum pattern in the sense that no permutation was significantly preferred over any other. A possible explanation for this is that it is the only statement in which the resource is wealth. In any case, the “proof of concept” has been demonstrated: Asymmetric zero-sum-like statement endorsements are not difficult to find.

The pattern in S6 (Rich-Poor) fits neither the ACP nor the ARF heuristic, but reveals another striking combination of unequal endorsements: Ab > Ba and Ab > aB, whereas bA > aB and bA > Ba. In short, gains for A paired with losses for B are chosen over their opposites in all four relevant comparisons. In the context of S6, this amounts to a greater belief in scenarios where the rich are getting richer while the poor get poorer, than in scenarios where the poor are getting richer while the rich get poorer. One way of interpreting this pattern is that gains have a greater potency for one consumer than for the other, so we shall refer to this pattern as the “asymmetric gains potency” (AGP) heuristic.

## Study 2

This study was designed to extend the investigation of asymmetric zero-sum beliefs in four respects. First, rating-scales were employed instead of two-alternative forced-choice formats, to surmount some of the limitations of those formats but also to ensure that the findings in Study 1 were not an artifact of task demands or response formats. Second, permutations of zero-sum-like propositions were arranged as a between-subjects factor, to ascertain whether the patterns of differential endorsement identified in Study 1 are replicated between as well as within participants, and also to enable all six comparisons among the permutations (Study 1 was limited to four comparisons). Third, we aimed to test the applicability of the heuristics identified in Study 1 by constructing zero-sum-like propositions intended to produce specific endorsement patterns.

Fourth, we wished to determine the generalizability of the findings in Study 1 and this study as well, at least across more than one English-speaking culture. In addition to testing a culture that is fairly similar to the USA, we also wished to sample from a culture that is less individualistic. Gelfand and Christakopoulou (1999) present arguments and evidence that members of less individualistic societies may be less inclined to hold zero-sum beliefs than those in strongly individualistic societies. Accordingly, we recruited samples from the USA, UK, and India. However, we wish to make it clear that an in-depth cross-cultural analysis is not within the scope of this paper. Instead, in this and Study 3 to follow, we are attempting to test our hypotheses on populations that are not “W.E.I.R.D.” (i.e., Western, Educated, Industrial, Rich, Developed; Henrich et al., 2010). That said, we recognize that Qualtrics panel samples are not random samples, and may or may not be representative of their referent populations.

Study 2 included four of the zero-sum-like propositions from Study 1 for purposes of replication, and four new propositions to test predictions of endorsement patterns. Permutations were arranged as between-subjects factors, and Likert rating scales were employed to measure endorsement. Study 2 also was embedded in a larger study involving individual-differences measures (described below, with findings reported in Smithson and Shou, 2016).

### Method

#### Participants

There were 496 completed surveys by adult participants (at least 18 years old), from each of the three countries, each with 50% males. The Qualtrics fee per participant was $8.17USD. The median and interquartile deviation for the time participants spent on the survey were 13.0 and 9.0 min, respectively. Participant data were excluded from the analyses if the participant spent less than 5 min on the survey, “flat-lined” responses, or did not answer “paying attention” questions correctly. The usable samples were as follows. The USA sample included 405 people, of whom 46.1% were males, and whose mean and standard deviation of age were 49.6 and 15.5, respectively. The UK sample included 426 people, of whom 49.3% were males, and whose mean and standard deviation of age were 46.6 and 18.2, respectively. The India sample included 452 people, of whom 51.8% were males, and whose mean and standard deviation of age were 37.5 and 17.2, respectively.

#### Materials and design

The study included two major parts, the zero-sum questions and a battery of items measuring the Big-5 personality factors (the BFI version: John et al., 1991), Social Dominance Orientation (Ho et al., 2012), and Competitive World View Perry et al. (2013). These individual difference measures are not discussed here, but their rationale and complete analysis may be found in Smithson and Shou (2016). The order of presentation of these two parts was counterbalanced. The zero-sum-like propositions were permuted in a between-subjects factor with four levels (as in Study 1; see Table 2). The overall design, therefore, was a 2 (orders) × 4 (permutations) factorial between-subjects experiment.

Table 5 displays the zero-sum-like propositions used in this study. Four of them (starred) are replications of propositions from Study 1. S1 and S5 were retained as exemplars of the ACP heuristic pattern in Study 1, S4 was retained because it exhibited the ARF pattern, and S6 because it revealed the AGP pattern. The new propositions were constructed for the following purposes: S2 and S3 were constructed to yield the ACP pattern, S7 was intended to produce the AGP pattern, and S8 was intended to provide a strict zero-sum pattern. Endorsement of the zero-sum-like propositions was measured by a 5-point Likert scale ranging from “strongly disagree” to “strongly agree.”

**Table 5:** **Study 2 zero-sum propositions**.

**Item**	**Label**	**Example (Ab)**
S1^*^	Work-personal	Devoting more time to work (A) takes time away from personal relationships (B).
S2	Distance-time	The longer the distance (A) from A to B, the more time (B) it takes to get from A to B.
S3	Eat-weigh	The more I eat (A), the more I weigh (B).
S4^*^	Best friend	If my best friend increases the attention they pay to someone else (B), they pay less attention to me (A).
S5^*^	Immigration-jobs	If the rate of immigration (A) is increased there will be fewer jobs (B) to go around.
S6^*^	Rich-poor	When the rich (A) get richer the poor (B) get poorer.
S7	Food-clothes	Spending more money on clothes (A) means there is less money to spend on food (B).
S8	Cloudy-sunny	The more hours of cloudy weather (A), the fewer hours of sunshine in a day (B).

* *Replicated statements from Study 1*.

#### Procedure

This study was conducted as an online experiment administered through a Qualtrics™ panel, subsequent to approval by The Australian National University Human Research Ethics Committee. Participants were randomly assigned to one of the eight conditions. After an introductory screen asking for informed consent, the participants completed the individual differences measures and zero-sum-like proposition questions in the order determined by the condition to which they were assigned. Participants were then asked to provide their country of origin, English language background, education level, age, and gender, after which they were thanked and debriefed via a closing information screen.

### Results

Ordinal logistic regression was used to analyze these data (using the cumulative logit model in the VGAM package in R, Yee, 2010). The dependent variable in all analyses is a categorical ordinal Likert scale, so the suitability of this technique and, likewise, the inappropriateness of normal-distribution-theory linear regression are self-evident. Owing to the large sample sizes, a significance criterion of 0.01 rather than the conventional 0.05 was adopted for model comparison purposes via likelihood-ratio tests. The primary comparisons of interest were between models restricted to main effects for country and statement permutation, and models that included moderator effects from country. The LL Ratio *p* column in Table 6 shows the *p*-values for the likelihood-ratio tests comparing these models for each of the eight statements (all *p*-values for main-effects models compared to intercept-only models were <0.001, so they are not reported here). S5 and S6 had moderation effects that significantly contributed to the models' goodness of fit and were retained in the final model. The other statements' final models are main-effects models.

**Table 6:** **Study 2 logistic regression model odds-ratio summaries**.

**Prop**.	**LL ratio *p***	**Sample**	**Odds-ratios**			
ACP + partial ARF			Ab > Ba	aB > bA	Ab > aB	Ba < bA
S1	0.010	All	4.61	2.39	2.01	
S2	0.035	All	4.08	1.74	1.38	1.69
S3	0.050	All	4.07	1.84	1.97	
S5	<0.001	USA	4.32	3.33	1.68	
		India	–	–	–	
		UK	6.01	2.66	2.05	
Partial ARF + Partial ACP			Ab > ba	aB > bA	Ba > bA	
S4	0.733	All	1.46	1.60	1.58	
S8	0.041	All	3.49	2.26	2.23	
	AGP		Ab > Ba	Ab > aB	bA > aB	bA > Ba
S6	0.003	USA	6.61	6.37	4.27	4.43
		India	12.94	5.53	5.32	12.44
		UK	4.02	2.57	3.43	5.37
S7	0.054	All	–	2.45	3.58	1.35

Exponentiated ordinal logistic regression coefficients have an odds-ratios interpretation that we utilize to present the results for comparisons such as aB with bA, rather than flooding this paper with tables of coefficients. In the first statement below, the main-effects model including the statement comparison and country terms can be written as

(2) $$\begin{array}{l} P\left(Y_{i}\leq j\right)=\alpha_{j}-\beta_{1}S_{Ba}-\beta_{2}S_{Ab}-\beta_{3}S_{bA}-\gamma_{1}C_{India}-\gamma_{2}C_{UK}\\ \;=\alpha_{j}-0.830S_{Ba}+0.699S_{Ab}-0.872S_{bA}\\ \;-0.148C_{India}-0.035C_{UK} \end{array}$$

The α_*j*_ is the threshold parameter for the *j*^th^ cumulative logits (effectively these are “nuisance” parameters, and in this model their values are α_1_ = −3.439, α_2_ = −1.277, α_3_ = −0.206, and α_4_ = 1.854), the *S*_*k*_ are dummy variables for the item permutations, and the *C*_*m*_ are dummy variables for the countries (note the negative coefficient signs, as the ordinal logistic model is parameterized in the VGAM package). For the item permutations, the reference category is the aB combination [Devoting less time to work (a) makes more time for personal relationships (B)]. The coefficient comparing aB and bA is 0.872 (*z* = 6.12, *p* < 0.001), and exp(0.872) = 2.39. Because of the negative coefficient signs, for any two-way split of our 5-point scale (e.g., 1−2 vs. 3−5), the odds of aB being rated in the upper part (e.g., a score of 3−5) of the scale is 2.39 times greater than the odds of bA being rated in the upper part of the scale, so we have aB > bA, which is part of the ACP pattern.

We now present the results for each of the eight propositions in turn. Table 6 displays the odds-ratio summaries for these propositions, grouped according to shared patterns of permutation effects. The histograms referred to throughout this section are in Appendix A in Supplementary Material.

*S1: Devoting more time to work (A) takes time away from personal relationships (B)*. The ACP pattern in the histograms is clear (aB > bA and Ab > Ba), and is backed up by the relevant odds-ratios. From equation (2), we have already seen that exponentiating the coefficient showing that aB > bA yields the odds-ratio exp(0.872) = 2.39. Comparing Ab with Ba requires exponentiating the appropriate combination of coefficients from equation (2) because aB is the referent category, thus, exp(0.830 + 0.699) = 4.61. Both comparisons thereby replicate the finding in Study 1. There is also one component of the ARF pattern (Ab > aB, odds-ratio exp(0.699) = 2.01). As this pattern crops up in subsequent results, we shall refer to it as a “partial ARF.” As indicated in Table 6, there is a borderline-significant country moderator effect. The UK and USA samples exhibit the pattern just described, whereas the India sample has only one component of ACP (Ab > Ba, odds-ratio = 2.83) and otherwise also fits this pattern.

*S2: The longer the distance (A) from A to B, the more time (B) it takes to get from A to B*. The model for this item is

(3) $$\begin{array}{l} P\left(Y_{i}\leq j\right)=\alpha_{j}-\beta_{1}S_{Ab}-\beta_{2}S_{aB}-\beta_{3}S_{Ba}-\gamma_{1}C_{India}-\gamma_{2}C_{UK}\\ \;=\alpha_{j}+0.878S_{Ab}+0.555S_{aB}-0.527S_{Ba}\\ \;+\;0.268C_{India}-0.669C_{UK} \end{array}$$

For the item permutations, the reference category is the bA combination. This proposition was predicted to exhibit the ACP pattern, and, as can be seen in the histograms, it does so. From equation (3) we have Ab > Ba and aB > bA [ACP, with odds-ratios exp(0.878 + 0.527) = 4.08 and exp (0.555) = 1.74, respectively], Ab > aB [partial ARF, odds-ratio exp(0.878 − 0.555) = 1.38), and Ba < bA (odds-ratio exp(0.527) = 1.69].

*S3: The more I eat (A), the more I weigh (B)*. The model for this item is

(4) $$\begin{array}{l} P\left(Y_{i}\leq j\right)=\alpha_{j}-\beta_{1}S_{Ab}-\beta_{2}S_{aB}-\beta_{3}S_{Ba}-\gamma_{1}C_{India}-\gamma_{2}C_{UK}\\ \;=\alpha_{j}+1.287S_{Ab}+0.608S_{aB}-0.116S_{Ba}\\ \;-\;0.120C_{India}-0.047C_{UK} \end{array}$$

For the item permutations, the reference category is the bA combination. This proposition also was predicted to exhibit the ACP pattern, and as the histograms demonstrate, it does. From equation (4) we have Ab > Ba and aB > bA [ACP, odds-ratios exp(1.287 + 0.116) = 4.07 and exp(0.608) = 1.84, respectively], and Ab > aB [partial ARF, odds-ratio exp(1.287 − 0.608) = 1.97].

*S4: If my best friend increases the attention they pay to me (A), they pay less attention to someone else (B)*. The model for this item is

(5) $$\begin{array}{l} P\left(Y_{i}\leq j\right)=\alpha_{j}-\beta_{1}S_{Ba}-\beta_{2}S_{bA}-\beta_{3}S_{Ab}-\gamma_{1}C_{India}-\gamma_{2}C_{UK}\\ \;=\alpha_{j}-0.011S_{Ba}-0.471S_{bA}-0.094S_{Ab}\\ \;+\;0.399C_{India}-0.061C_{UK} \end{array}$$

For the item permutations, the reference category is the aB combination. This proposition produced the ARF pattern in Study 1, but in this study it only partially does this, while exhibiting another distinctive pattern. In the histograms, it is apparent that aB > bA, Ab > bA, and Ba > bA. From equation (5) we have odds-ratios exp(0.471) = 1.60, exp(0.471 − 0.094) = 1.46, and exp(0.471 − 0.011) = 1.58, respectively. There are no other inequalities in endorsement among the four permutations. This pattern is a combination of ARF and ACP, which we shall denote by “partial ARF + partial ACP.”

*S5: If the rate of immigration (A) is increased there will be fewer jobs (B) to go around*. In Study 1 this proposition produced an ACP pattern, and the histograms suggest that it does so here too. However, the findings are somewhat more complex. The model for this item is

(6) $$\begin{array}{l} P\left(Y_{i}\leq j\right)=\alpha_{j}-\beta_{1}S_{Ab}-\beta_{2}S_{aB}-\beta_{3}S_{Ba}-\gamma_{1}C_{India}-\gamma_{2}C_{UK}\\ \;-\;\delta_{11}S_{Ab}C_{India}-\delta_{12}S_{Ab}C_{UK}-\delta_{21}S_{aB}C_{India}\\ \;-\;\delta_{22}S_{aB}C_{UK}-\delta_{31}S_{Ba}C_{India}-\delta_{32}S_{Ba}C_{UK}\\ \;=\;\alpha_{j}+1.721S_{Ab}+1.202S_{aB}+0.258S_{Ba}\\ \;+\;1.304C_{India}+0.283C_{UK}-1.780S_{Ab}C_{India}\\ \;+\;0.023S_{Ab}C_{UK}-\;1.113S_{aB}C_{India}-0.223S_{aB}C_{UK}\\ \;+\;0.095S_{Ba}C_{India}-0.308S_{Ba}C_{UK} \end{array}$$

For the item permutations, the reference category is the bA combination. The USA and UK samples exhibit the full ACP pattern (replicating Study 1), as well as Ab > aB (partial ARF). In the USA sample, the odds-ratios for Ab > Ba and aB > bA (ACP) are exp(1.721 − 0.258) = 4.32 and exp(1.202) = 3.33, respectively; while in the UK sample these odds-ratios are exp(1.721 − 0.258 + 0.023 + 0.308) = 6.01 and exp(1.202 − 0.223) = 2.66. However, the India sample shows no significant differences in endorsement of any permutations, i.e., a consistent zero-sum pattern. The net Ab, aB, and Ba coefficients for the Indian sample are 1.721 − 1.780 = −0.059 (*z* = −0.25, *p* > 0.05), 1.202 − 1.113 = 0.089 (*z* = 0.38, *p* > 0.05), and 0.258 + 0.095 = 0.353 (*z* = 1.49, *p* > 0.05), respectively.

*S6: When the rich (A) get richer the poor (B) get poorer*. In Study 1, this proposition revealed the AGP pattern described earlier. In the histograms, this pattern is quite evidently replicated, despite country moderator effects. The model for this item is

(7) $$\begin{array}{l} P\left(Y_{i}\leq j\right)=\alpha_{j}-\beta_{1}S_{Ab}-\beta_{2}S_{aB}-\beta_{3}S_{Ba}-\gamma_{1}C_{India}\\ \;-\;\gamma_{2}C_{UK}-\delta_{11}S_{Ab}C_{India}-\delta_{12}S_{Ab}C_{UK}\\ \;-\;\delta_{21}S_{aB}C_{India}-\delta_{22}S_{aB}C_{UK}-\delta_{31}S_{Ba}C_{India}\\ \;-\;\delta_{32}S_{Ba}C_{UK}\\ \;=\;\alpha_{j}+0.400S_{Ab}-1.451S_{aB}-1.489S_{Ba}\\ \;+\;0.753C_{India}+0.318C_{UK}-0.361S_{Ab}C_{India}\\ \;-\;0.688S_{Ab}C_{UK}-0.220S_{aB}C_{India}+0.219S_{aB}C_{UK}\\ \;-\;1.032S_{Ba}C_{India}-\;0.191S_{Ba}C_{UK} \end{array}$$

For the item permutations, the reference category is the bA combination. We have the full AGP pattern, Ab > Ba, Ab > aB, bA > aB, and bA > Ba in the three samples. The USA sample odds-ratios are exp(0.400 + 1.489) = 6.61, exp(0.400 + 1.451) = 6.37, exp(1.451) = 4.27, and exp(1.489) = 4.43, respectively. The UK sample odds-ratios are exp(0.400 + 1.489 − 0.688 + 0.191) = 4.02, exp(0.400 + 1.451 − 0.688 − 0.219) = 2.57, exp(1.451 − 0.219) = 3.43, and exp(1.489 + 0.191) = 5.37, respectively. And the India sample odds-ratios are exp(0.400 + 1.489 − 0.361 + 1.032) = 12.94, exp(0.400 + 1.451 − 0.361 + 0.220) = 5.53, exp(1.451 + 0.220) = 5.32, and exp(1.489 + 1.032) = 12.44, respectively.

*S7: Spending more money on food (A) means there is less money to spend on clothes (B)*. The model for this item is

(8) $$\begin{array}{l} P\left(Y_{i}\leq j\right)=\alpha_{j}-\beta_{1}S_{Ba}-\beta_{2}S_{Ab}-\beta_{3}S_{bA}-\gamma_{1}C_{India}-\gamma_{2}C_{UK}\\ \;=\;\alpha_{j}+0.976S_{Ba}+0.895S_{Ab}+1.276S_{bA}\\ \;-\;0.813C_{India}+0.008C_{UK} \end{array}$$

For the item permutations, the reference category is the aB combination. This proposition was predicted to produce the AGP pattern. Inspection of the histograms reveals that the results do not quite fit this pattern. Instead, from equation (8) we have Ab > aB [odds-ratio exp(0.895) = 2.45), bA > aB (odds-ratio exp(1.276) = 3.58), and bA > Ba (odds-ratio exp(1.276 − 0.976) = 1.35], but not Ab > Ba (therefore, not quite AGP). It also is clear that all permutations are more strongly endorsed than aB.

*S8: The more hours of cloudy weather (A), the fewer hours of sunshine in a day (B)*. The model for this item is

(9) $$\begin{array}{l} P\left(Y_{i}\leq j\right)=\alpha_{j}-\beta_{1}S_{Ab}-\beta_{2}S_{aB}-\beta_{3}S_{bA}-\gamma_{1}C_{India}-\gamma_{2}C_{UK}\\ \;=\alpha_{j}+0.445S_{Ab}+0.010S_{aB}-0.804S_{bA}\\ \;-\;0.014C_{India}-0.107C_{UK} \end{array}$$

For the item permutations, the reference category is the Ba combination. This proposition was predicted to produce a consistent zero-sum pattern, i.e., with no unequal endorsements among permutations. The histograms show that this prediction has proved incorrect. Instead, from equation (9) we have the partial ARF + partial ACP pattern found in S4: Ab > bA [odds-ratio exp(0.445 + 0.804) = 3.49], aB > bA [odds-ratio exp(0.010 + 0.804) = 2.26], and Ba > bA [odds-ratio exp(0.804) = 2.23].

### Study 2 discussion

Study 2 partly replicated and extended the findings from Study 1. S1 and S5 both were expected to yield the ACP pattern, which they did for the USA and UK samples although not for the Indian sample. S1 and S5 also exhibited a partial ARF pattern, which Study 1 was incapable of detecting, and which was echoed in S2 and S3. We shall denote this pattern by “partial ARF + ACP.” S4 partially replicated the ARF finding from Study 1, yielding the partial ARF + partial ACP pattern for all three samples. Finally, S6 replicated the AGP pattern from Study 1 for all three samples. Overall, the evidence suggests that most of the heuristic patterns identified in Study 1 may generalize across English-speaking cultures.

Turning now to the predictions regarding the new items, both S2 and S3 were predicted to have the ACP pattern and yielded partial ARF + ACP. The other two predictions were disconfirmed. S7 did not produce the AGP pattern, nor did S8 yield a strict zero-sum result. Instead, S8 showed partial ARF + partial ACP and S7 showed an inverted version of the partial ARF + ACP pattern. Again, these patterns held for all three samples, indicating English-speaking cross-cultural generality.

New and consistent outcomes in Study 2 are the partial ARF + partial ACP and partial ARF + ACP patterns. Study 1 was incapable of revealing these patterns, so they are very likely due to the additional comparisons among permutations made possible in Study 2. These combinations were observed in all of the statements except for S6 and S7. None of the statements exhibited ACP on its own, but always in conjunction with a partial or full ARF pattern. The greatest consistency of this combination was found for the USA and UK samples, but it also occurred in the Indian sample for S2, S3, S4, and S8. The fact that this pattern was obtained even for the objectively zero-sum S8 indicates its pervasiveness in people's interpretations of zero-sum-like statements.

## Study 3

Study 3 was designed to test the generality of the results from Studies 1 and 2 in two respects. First, the rating scale was expanded from 5 to 7 bins, to test whether any of the patterns identified in the previous studies might be artifacts of the scale response format. To do this, six of the Study 2 statements were retained for Study 3 and adult samples were obtained again from the USA, UK, and India. Second, cross-cultural generality was further tested by translating the items into Chinese and obtaining a sample of adult Chinese responses. The goal here was to explore whether the same patterns would emerge when the zero-sum-like statements are expressed in a language unrelated to English. Again, the goal here is not in-depth cross-cultural comparisons, but simply to replicate and extend the findings of the preceding studies, while gaining preliminary indications of whether language makes a difference.

### Method

#### Participants

As in the preceding studies, participant data were excluded from the analyses if the participant spent less than 5 min on the survey, “flat-lined” responses, or did not answer “paying attention” questions correctly. However, in this study we recruited participants until we had attained very close to an even gender split and 500 participants in each country fulfilling these criteria. The Qualtrics fee per participant was $8.50USD. The median and interquartile deviation for the time participants spent on the survey were 13.0 min and 10.0 min, respectively. The usable USA sample included 498 people (out of 510 total), whose mean and standard deviation of age were 45.8 and 15.6, respectively. The UK sample included 494 people (out of 511 total), whose mean and standard deviation of age were 51.6 and 14.4, respectively. The India sample included 503 people (out of 513 total), whose mean and standard deviation of age were 36.4 and 14.2, respectively. The China sample included 496 people (out of 506 total), whose mean and standard deviation of age were 32.3 and 7.8, respectively.

#### Materials, design, and procedure

As in Study 2, this study included two major parts, the zero-sum questions and a set of individual differences covariates, the findings for which are reported in Smithson and Shou (2016). The covariates consisted of subscales of two psychopathy inventories, the LSRP (Levenson et al., 1995), and the SRP II (Lester et al., 2012). The order of presentation of the zero-sum-like items and psychopathy inventories was counterbalanced in each of the four samples.

Table 7 displays the zero-sum-like propositions used in this study. Two of them (S2 and S3) are replicates of statements from Study 1 that were not retested in Study 2. Four others (S1, S4, S5, and S6) are replicates of items from Study 2 that also were employed in Study 1. The remaining two (S7 and S8) are new items, designed to test hypotheses regarding marginalizing racism (Smithson et al., 2015). They are analyzed here because they also were predicted to exhibit the AGP pattern. Endorsement of the zero-sum-like propositions was measured by a 7-point Likert scale ranging from “strongly disagree” to “strongly agree.”

**Table 7:** **Study 3 zero-sum propositions**.

**Item**	**Label**	**Example (Ab)**
S1^*^	Work-personal	Devoting more time to work (A) takes time away from personal relationships (B).
S2^#^	Friends-family	Spending more time with friends (A) takes time away from family (B).
S3^#^	Investment	Investing more money in one venture (A) means there is less for the others (B).
S4^*^	Best friend	If my best friend increases the attention they pay to someone else (B), they pay less attention to me (A).
S5^*^	Immigration-jobs	If the rate of immigration (A) is increased there will be fewer jobs (B) to go around.
S6^*^	Rich-poor	When the rich (A) get richer the poor (B) get poorer.
S7	Al-Bayati	: Consider Hama Al-Bayati, who immigrated to the U.S.A. (U.K., India) 5 years ago from Iraq. The more “Iraqi” (A) he is, the less “American” (“British,” “Indian”) (B) he will be.
S8	Al-Husseni	: Consider Ali Al-Husseni, who immigrated to Germany 5 years ago from Iraq. The more “Iraqi” (A) he is, the less “German” (B) he will be.

# *Replicated statements from Study 1*.

* *Replicated statements from Studies 1 and 2*.

The Chinese version of the online survey was translated into simplified Chinese and back-translated into English by two native-speakers of Chinese who are fluent in English. The S7 and S8 items are not relevant in Chinese society because immigration to China is rare, so substitutes were constructed that refer to a Chinese citizen migrating from one province to another. The substitute for statement 1 had the person immigrating to the home province of the respondent, while the substitute for statement 2 had the person immigrating to another province (Xinjiang).

This study was conducted as an online experiment administered through a Qualtrics™ panel, subsequent to approval by The Australian National University Human Research Ethics Committee. The procedure was identical to that in Study 2.

### Results

Ordinal logistic regression was employed for analyzing the data in the same way that it was in Study 2. The same criteria for model comparison also were used. The LL Ratio *p* column in Table 8 displays the *p*-values for the likelihood-ratio tests between main-effects and moderator-effects models (all *p*-values for main-effects models compared to intercept-only models were <0.001, so they are not reported here). Moderator-effects models are the final models for statements 1, 5, and 6, as in Study 2, but also for statements 2 and 4 (unlike in Study 2), and 7 and 8.

**Table 8:** **Study 3 logistic regression model odds-ratio summaries**.

**Prop**.	**LL ratio *p***	**Sample**	**Odds-ratios**			
ACP + partial ARF			Ab > Ba	aB > bA	Ab > aB	Ba < bA
S1	<0.001	USA	6.82	1.73	2.20	
		UK	8.58	6.89	3.35	
		India	2.16	2.25	1.36	
		China	4.62	2.64	3.42	
S2	0.003	USA	2.39	4.95		
		UK	2.14	3.22		
		India	2.16	6.05		
		China	2.53	1.88		
S5	<0.001	USA	6.05	5.31		
		UK	12.68	3.42	Ba > bA	
		India			1.68	
		China	2.08	1.41		
S3	0.456	All				
Partial ARF + Partial ACP			Ab > ba	aB > bA	Ba > bA	
S4	0.009	USA	2.36	1.80	1.79	
		UK	1.82	2.18	2.08	
		India	2.59	1.72	1.86	
		China		2.27	1.73	
	AGP		Ab > Ba	Ab > aB	bA > aB	bA > Ba
S6	<0.001	USA	2.75	3.39	5.75	4.66
		UK	9.58	8.67	8.76	9.68
		India	12.43	9.68	10.80	13.87
		China	6.62	3.46	4.01	7.69
S7	<0.001	USA	2.20	2.48	2.29	2.03
		UK	11.13	10.28	11.25	12.18
		India	4.85	2.25		2.46
		China	1.77	1.92	2.46	2.48
S8	<0.001	USA	2.14	2.92	2.41	1.77
		UK	10.59	10.07	7.10	7.46
		India	4.71	4.57	2.46	2.53
		China	1.75	2.66	2.27	1.49

*Study 3 S1: Devoting more time to work (A) takes time away from personal relationships (B)*. Appendix B in Supplementary Material displays the histograms for the four permutations of S1. The model for this item is

(10) $$\begin{array}{l} P\left(Y_{i}\leq j\right)=\alpha_{j}-\beta_{1}S_{Ba}-\beta_{2}S_{Ab}-\beta_{3}S_{bA}-\gamma_{1}C_{UK}-\gamma_{2}C_{Ind}\\ \;-\;\gamma_{3}C_{Ch}-\delta_{11}S_{Ba}C_{UK}-\delta_{12}S_{Ba}C_{Ind}-\delta_{13}S_{Ba}C_{Ch}\\ \;-\;\delta_{21}S_{Ab}C_{UK}-\delta_{22}S_{Ab}C_{Ind}-\delta_{23}S_{Ab}C_{Ch}\\ \;-\;\delta_{31}S_{bA}C_{UK}-\delta_{32}S_{bA}C_{Ind}-\delta_{33}S_{bA}C_{Ch}\\ \;=\;\alpha_{j}-1.13S_{Ba}+0.79S_{Ab}-0.55S_{bA}+0.71C_{UK}\\ \;-\;0.77C_{Ind}-0.60C_{Ch}-0.65S_{Ba}C_{UK}\\ \;+\;1.63S_{Ba}C_{Ind}+0.83S_{Ba}C_{Ch}-0.42S_{Ab}C_{UK}\\ \;+\;0.48S_{Ab}C_{Ind}-0.44S_{Ab}C_{Ch}-1.38S_{bA}C_{UK}\\ \;+\;0.26S_{bA}C_{Ind}-0.19S_{bA}C_{Ch} \end{array}$$

For the item permutations, the reference category is the aB combination. Equation (10) shows that there are country moderator effects to take into account. Nevertheless, all four samples follow the partial ARF + ACP pattern, i.e., the ACP pattern [Ab > Ba and aB > bA: USA odds-ratios exp(1.13 + 0.79) = 6.82 and exp(0.55) = 1.73, respectively; UK odds-ratios exp(1.13 + 0.79 + 0.65 − 0.42) = 8.58 and exp(0.55 + 1.38) = 6.89, respectively; India odds-ratios exp(1.13 + 0.79 − 1.63 + 0.48) = 2.16 and exp(0.55 + 0.26) = 2.25, respectively; and China odds-ratios exp(1.13 + 0.79 − 0.83 + 0.44) = 4.62 and exp(0.55 + 0.42) = 2.64, respectively], and a partial ARF pattern [Ab > aB: USA odds-ratio exp(0.79) = 2.20, UK odds-ratio exp(0.79 + 0.42) = 3.35, India odds-ratio exp(0.79 − 0.48) = 1.36, and China odds-ratio exp(0.79 + 0.44) = 3.42], thereby replicating these findings in Study 2 and extending them to the Chinese sample.

*Study 3 S2: Spending more time with friends (A) takes time away from family (B)*. Study 1 suggested that this proposition would exhibit the ACP pattern, and the histograms in Appendix B in Supplementary Material suggest that it does this. The model for this item is

(11) $$\begin{array}{l} P\left(Y_{i}\leq j\right)=\;\alpha_{j}-\beta_{1}S_{Ab}-\beta_{2}S_{Ba}-\beta_{3}S_{aB}-\gamma_{1}C_{UK}-\gamma_{2}C_{Ind}\\ \;-\;\gamma_{3}C_{Ch}-\delta_{11}S_{Ab}C_{UK}-\delta_{12}S_{Ab}C_{Ind}-\delta_{13}S_{Ab}C_{Ch}\\ \;-\;\delta_{21}S_{Ba}C_{UK}-\delta_{22}S_{Ba}C_{Ind}-\delta_{23}S_{Ba}C_{Ch}\\ \;-\;\delta_{31}S_{aB}C_{UK}-\delta_{32}S_{aB}C_{Ind}-\delta_{33}S_{aB}C_{Ch}\\ \;=\alpha_{j}+1.31S_{Ab}+0.44S_{Ba}+1.60S_{aB}+0.15C_{UK}\\ \;-\;0.08C_{Ind}+0.51C_{Ch}-0.38S_{Ab}C_{UK}\\ \;+\;0.14S_{Ab}C_{Ind}-0.01S_{Ab}C_{Ch}\;-\;0.27S_{Ba}C_{UK}\\ \;+\;0.24S_{Ba}C_{Ind}\;+\;0.07S_{Ba}C_{Ch}\\ \;-\;0.43S_{aB}C_{UK}+0.20S_{aB}C_{Ind}-0.97S_{aB}C_{Ch} \end{array}$$

For the item permutations, the reference category is the bA combination. From equation (11), all four samples follow the ACP pattern [Ab > Ba and aB > bA: USA odds-ratios exp(1.31 − 0.44) = 2.39 and exp(1.60) = 4.95, respectively; UK odds-ratios exp(1.31 − 0.44 − 0.38 + 0.27) = 2.14 and exp(1.60 − 0.43) = 3.22, respectively; India odds-ratios exp(1.31 − 0.44 + 0.14 − 0.24) = 2.16 and exp(1.60 + 0.20) = 6.05, respectively; and China odds-ratios exp(1.31 − 0.44 − 0.01 + 0.07) = 2.53 and exp(1.60 − 0.97) = 1.88, respectively], thereby replicating the Study 1 result and extending it to the UK, India, and China.

*Study 3 S3: Investing more money in one venture (A) means there is less for the others (B)*. The model for this item is

(12) $$\begin{array}{l} P\left(Y_{i}\leq j\right)=\alpha_{j}-\beta_{1}S_{Ab}-\beta_{2}S_{aB}-\beta_{3}S_{Ba}-\gamma_{1}C_{UK}\\ \;-\;\gamma_{2}C_{Ind}-\gamma_{3}C_{Ch}\\ \;=\alpha_{j}-0.21S_{Ab}-0.48S_{aB}-0.24S_{Ba}\\ \;+\;0.17C_{UK}+0.69C_{Ind}+0.80C_{Ch} \end{array}$$

For the item permutations, the reference category is the bA combination. The Study 1 finding for this proposition indicated no significantly unequal endorsement of any of the four permutations. Although a log-likelihood ratio-test comparing a model with only main effects for country with one that adds main effects for permutation is significant (*p* < 0.001), the only notable difference is bA > aB (odds-ratio exp(0.48) = 1.62). All other comparisons have odds-ratios of less than 1.3.

*Study 3 S4: If my best friend increases the attention they pay to someone else (B), they pay less attention to me (A)*. The model for this item is

(13) $$\begin{array}{l} P\left(Y_{i}\leq j\right)=\alpha_{j}-\beta_{1}S_{Ba}-\beta_{2}S_{bA}-\beta_{3}S_{Ab}-\;\gamma_{1}C_{UK}-\gamma_{2}C_{Ind}\\ \;-\;\gamma_{3}C_{Ch}-\delta_{11}S_{Ba}C_{UK}-\delta_{12}S_{Ba}C_{Ind}-\delta_{13}S_{Ba}C_{Ch}\\ \;-\;\delta_{21}S_{bA}C_{UK}-\delta_{22}S_{bA}C_{Ind}-\delta_{23}S_{bA}C_{Ch}\\ \;-\;\delta_{31}S_{Ab}C_{UK}-\delta_{32}S_{Ab}C_{Ind}-\delta_{33}S_{Ab}C_{Ch}\\ \;=\;\alpha_{j}-0.01S_{Ba}-0.59S_{bA}+0.27S_{Ab}-0.01C_{UK}\\ \;+\;0.34C_{Ind}+0.58C_{Ch}+\;0.04S_{Ba}C_{UK}\\ \;-\;0.09S_{Ba}C_{Ind}-0.26S_{Ba}C_{Ch}-0.19S_{bA}C_{UK}\\ \;+\;0.05S_{bA}C_{Ind}-0.23S_{bA}C_{Ch}-0.45S_{Ab}C_{UK}\\ \;-\;0.14S_{Ab}C_{Ind}-1.22S_{Ab}C_{Ch} \end{array}$$

For the item permutations, the reference category is the aB combination. Study 2 indicated that this proposition has the partial ARF + partial ACP pattern Ab > bA, aB > bA, and Ba > bA, and and the histograms in Appendix B in Supplementary Material suggest the same pattern. From equation (13), three of the samples display this pattern [USA odds-ratios exp(0.59 + 0.27) = 2.36, exp(0.59) = 1.80, and exp(0.59 − 0.01) = 1.79, respectively; UK odds-ratios exp(0.59 + 0.27 + 0.19 − 0.45) = 1.82, exp(0.59 + 0.19) = 2.18, and exp(0.59 − 0.01 + 0.19 − 0.04) = 2.08, respectively; and India odds-ratios exp(0.59 + 0.27 − 0.05 + 0.14) = 2.59, exp(0.59 − 0.05) = 1.72, and exp(0.59 − 0.01 − 0.05 + 0.09) = 1.86, respectively]. China partially replicates it with an ARF pattern (aB > bA and Ba > bA: odds-ratios exp(0.59 + 0.23) = 2.27 and exp(0.59 − 0.01 + 0.23 − 0.26) = 1.73, respectively).

*Study 3 S5: If the rate of immigration (A) is increased there will be fewer jobs (B) to go around*. In Study 2 this proposition produced the partial ARF + ACP pattern (except for the India sample), and the histograms in Appendix B in Supplementary Material suggest that it does so here. The model for this item is

(14) $$\begin{array}{l} P\left(Y_{i}\leq j\right)=\alpha_{j}-\beta_{1}S_{Ab}-\beta_{2}S_{aB}-\beta_{3}S_{Ba}-\gamma_{1}C_{UK}-\gamma_{2}C_{Ind}\\ \;-\;\gamma_{3}C_{Ch}-\delta_{11}S_{Ab}C_{UK}-\delta_{12}S_{Ab}C_{Ind}-\delta_{13}S_{Ab}C_{Ch}\\ \;-\;\delta_{21}S_{aB}C_{UK}-\delta_{22}S_{aB}C_{Ind}-\delta_{23}S_{aB}C_{Ch}\\ \;-\;\delta_{31}S_{Ba}C_{UK}-\delta_{32}S_{Ba}C_{Ind}-\delta_{33}S_{Ba}C_{Ch}\\ \;=\;\alpha_{j}+1.96S_{Ab}+1.67S_{aB}+0.16S_{Ba}+0.82C_{UK}\\ \;+\;1.63C_{Ind}+0.86C_{Ch}-0.09S_{Ab}C_{UK}\\ \;-\;1.70S_{Ab}C_{Ind}-1.59S_{Ab}C_{Ch}-0.44S_{aB}C_{UK}\\ \;-\;1.55S_{aB}C_{Ind}-1.33S_{aB}C_{Ch}-0.83S_{Ba}C_{UK}\\ \;+\;0.36S_{Ba}C_{Ind}-0.52S_{Ba}C_{Ch} \end{array}$$

For the item permutations, the reference category is the bA combination. Taking country moderator effects into account, the USA, UK, and China samples exhibit the full ACP pattern [Ab > Ba and aB > bA: USA odds-ratios exp(1.96 − 0.16) = 6.05 and exp(1.67) = 5.31, respectively; UK odds-ratios exp(1.96 − 0.09 − 0.16 + 0.83) = 12.68 and exp(1.67 − 0.44) = 3.42, respectively; and China odds-ratios exp(1.96 − 0.16 − 1.59 + 0.52) = 2.08 and exp(1.67 − 1.33) = 1.41, respectively]. The USA and UK samples also have the partial ARF pattern, Ab > aB [USA odds-ratio exp(1.96 − 1.67) = 1.34 and UK odds-ratio exp(1.96 − 1.67 − 0.09 + 0.44) = 1.90). However, the India sample shows only one inequality in endorsement: Ba > bA (odds-ratio exp(0.16 + 0.36) = 1.68].

*Study 3 S6: When the rich (A) get richer the poor (B) get poorer*. In Studies 1 and 2, this proposition yielded the AGP pattern. The histograms in Appendix B in Supplementary Material show this pattern clearly. The model for this item is

(15) $$\begin{array}{l} P\left(Y_{i}\leq j\right)=\alpha_{j}-\beta_{1}S_{Ab}-\beta_{2}S_{aB}-\beta_{3}S_{Ba}-\gamma_{1}C_{UK}-\gamma_{2}C_{Ind}\\ \;-\;\gamma_{3}C_{Ch}-\delta_{11}S_{Ab}C_{UK}-\delta_{12}S_{Ab}C_{Ind}-\delta_{13}S_{Ab}C_{Ch}\\ \;-\;\delta_{21}S_{aB}C_{UK}-\delta_{22}S_{aB}C_{Ind}-\delta_{23}S_{aB}C_{Ch}\\ \;-\;\delta_{31}S_{Ba}C_{UK}-\delta_{32}S_{Ba}C_{Ind}-\delta_{33}S_{Ba}C_{Ch}\\ \;=\;\alpha_{j}-0.53S_{Ab}-1.75S_{aB}-1.54S_{Ba}+0.38C_{UK}\\ \;+\;0.69C_{Ind}+0.24C_{Ch}+0.52S_{Ab}C_{UK}\\ \;+\;0.42S_{Ab}C_{Ind}+0.38S_{Ab}C_{Ch}-0.42S_{aB}C_{UK}\\ \;-\;0.63S_{aB}C_{Ind}+0.36S_{aB}C_{Ch}-0.73S_{Ba}C_{UK}\\ \;-\;1.09S_{Ba}C_{Ind}-0.50S_{Ba}C_{Ch} \end{array}$$

For the item permutations, the reference category is the bA combination. Taking country moderator effects into account, we have the full AGP pattern for all four samples: Ab > Ba and Ab > aB [USA odds-ratios exp(−0.53 + 1.54) = 2.75 and exp(−0.53 + 1.75) = 3.39, respectively; UK odds-ratios = exp(−0.53 + 1.54 + 0.52 + 0.73) = 9.58 and exp(−0.53 + 1.75 + 0.52 + 0.42) = 8.67, respectively; India odds-ratios exp(−0.53 + 1.54 + 0.42 + 1.09) = 12.43 and exp(−0.53 + 1.75 + 0.42 + 0.63) = 9.68, respectively, and China odds-ratios exp(−0.53 + 1.54 + 0.38 + 0.50) = 6.62 and exp(−0.53 + 1.75 + 0.38 − 0.36) = 3.46, respectively]; and bA > aB and bA > Ba [USA odds-ratios exp (1.75) = 5.75 and exp (1.54) = 4.66, respectively; UK odds-ratios exp(1.75 + 0.42) = 8.76 and exp (1.54 + 0.73) = 9.68, respectively; India odds-ratios exp (1.75 + 0.63) = 10.80 and exp (1.54 + 1.09) = 13.87, respectively; and China odds-ratios exp (1.75 − 0.36) = 4.01 and exp (1.54 + 0.50) = 7.69, respectively].

*Study 3 S7: The more “Iraqi”(A) he is, the less “Own Country”(B) he will be*. As hypothesized, the histograms in Appendix B in Supplementary Material suggest the AGP pattern. The model for this item is

(16) $$\begin{array}{l} P\left(Y_{i}\leq j\right)=\alpha_{j}-\beta_{1}S_{Ab}-\beta_{2}S_{Ba}-\beta_{3}S_{aB}-\gamma_{1}C_{UK}-\gamma_{2}C_{Ind}\\ \;-\;\gamma_{3}C_{Ch}-\delta_{11}S_{Ab}C_{UK}-\delta_{12}S_{Ab}C_{Ind}-\delta_{13}S_{Ab}C_{Ch}\\ \;-\;\delta_{21}S_{Ba}C_{UK}-\delta_{22}S_{Ba}C_{Ind}-\delta_{23}S_{Ba}C_{Ch}\\ \;-\;\delta_{31}S_{aB}C_{UK}-\delta_{32}S_{aB}C_{Ind}-\delta_{33}S_{aB}C_{Ch}\\ \;=\;\alpha_{j}+0.08S_{Ab}-0.71S_{Ba}-0.83S_{aB}+1.44C_{UK}\\ \;+\;0.44C_{Ind}+1.01C_{Ch}-0.17S_{Ab}C_{UK}\\ \;+\;0.60S_{Ab}C_{Ind}-0.42S_{Ab}C_{Ch}-1.79S_{Ba}C_{UK}\\ \;-\;0.19S_{Ba}C_{Ind}-0.20S_{Ba}C_{Ch}-1.59S_{aB}C_{UK}\\ \;+\;0.70S_{aB}C_{Ind}-0.16S_{aB}C_{Ch} \end{array}$$

For the item permutations, the reference category is the bA combination. Taking country moderator effects into account, we have the full AGP pattern for all four samples with one exception in the Indian sample: Ab > Ba and Ab > aB [USA odds-ratios exp(0.08 + 0.71) = 2.20 and exp(0.08 + 0.83) = 2.48, respectively; UK odds-ratios exp(0.08 + 0.71 − 0.17 + 1.79) = 11.13 and exp(0.08 + 0.83 − 0.17 + 1.59) = 10.28, respectively; India odds-ratios exp(0.08 + 0.71 + 0.60 + 0.19) = 4.85 and exp(0.08 + 0.83 + 0.60 − 0.70) = 2.25, respectively, and China odds-ratios exp(0.08 + 0.71 − 0.42 + 0.20) = 1.77 and exp(0.08 + 0.83 − 0.42 + 0.16) = 1.92, respectively]; and bA > aB and bA > Ba [USA odds-ratios exp(0.83) = 2.29 and exp(0.71) = 2.03, respectively; UK odds-ratios exp(0.83 + 1.59) = 11.25 and exp(0.71 + 1.79) = 12.18, respectively; India odds-ratios exp(0.83 − 0.70) = 1.14, non-significant, and exp(0.71 + 0.19) = 2.46, respectively; and China odds-ratios exp(0.83 + 0.16) = 2.69 and exp(0.71 + 0.20) = 2.48, respectively].

*Study 3 S8: The more “Iraqi”(A) he is, the less “German”(B) he will be*. As hypothesized, the histograms in Appendix B in Supplementary Material suggest the AGP pattern. The model for this item is

(17) $$\begin{array}{l} P\left(Y_{i}\leq j\right)=\alpha_{j}-\beta_{1}S_{Ab}-\beta_{2}S_{Ba}-\beta_{3}S_{aB}-\gamma_{1}C_{UK}-\gamma_{2}C_{Ind}\\ \;-\;\gamma_{3}C_{Ch}-\delta_{11}S_{Ab}C_{UK}-\delta_{12}S_{Ab}C_{Ind}-\delta_{13}S_{Ab}C_{Ch}\\ \;-\;\delta_{21}S_{Ba}C_{UK}-\delta_{22}S_{Ba}C_{Ind}-\delta_{23}S_{Ba}C_{Ch}\\ \;-\;\delta_{31}S_{aB}C_{UK}-\delta_{32}S_{aB}C_{Ind}-\delta_{33}S_{aB}C_{Ch}\\ \;=\;\alpha_{j}+0.19S_{Ab}-0.57S_{Ba}-0.88S_{aB}+1.07C_{UK}\\ \;+\;0.54C_{Ind}+0.49C_{Ch}+0.16S_{Ab}C_{UK}\\ \;+\;0.43S_{Ab}C_{Ind}-0.03S_{Ab}C_{Ch}-1.44S_{Ba}C_{UK}\\ \;-\;0.36S_{Ba}C_{Ind}+0.17S_{Ba}C_{Ch}-1.08S_{aB}C_{UK}\\ \;-\;0.02S_{aB}C_{Ind}+0.06S_{aB}C_{Ch} \end{array}$$

For the item permutations, the reference category is the bA combination. Taking country moderator effects into account, we have the full AGP pattern for all four samples: Ab > Ba and Ab > aB [USA odds-ratios exp(0.19 + 0.57) = 2.14 and exp(0.19 + 0.88) = 2.92, respectively; UK odds-ratios = exp(0.19 + 0.57 + 0.16 + 1.44) = 10.59 and exp(0.19 + 0.88 + 0.16 + 1.08) = 10.07, respectively; India odds-ratios exp(0.19 + 0.57 + 0.43 + 0.36) = 4.71 and exp(0.19 + 0.88 + 0.43 + 0.02) = 4.57, respectively, and China odds-ratios exp(0.19 + 0.57 − 0.03 − 0.17) = 1.75 and exp(0.19 + 0.88 − 0.03 − 0.06) = 2.66, respectively]; and bA > aB and bA > Ba [USA odds-ratios exp(0.88) = 2.41 and exp(0.57) = 1.77, respectively; UK odds-ratios exp(0.88 + 1.08) = 7.10 and exp(0.57 + 1.44) = 7.46, respectively; India odds-ratios exp (0.88 + 0.02) = 2.46 and exp(0.57 + 0.36) = 2.53, respectively; and China odds-ratios exp(0.88 − 0.06) = 2.27 and exp(0.57 − 0.17) = 1.49, respectively].

### Study 3 discussion

Study 3 replicated two additional findings from Study 1 and extended them to the UK, India, and China. S2 and S3 exhibited the patterns found for them in Study 1 (ACP and strict zero-sum, respectively) in the samples from all four countries. Study 3 replicated the Study 2 findings in the American, British, and Indian samples for S4, S5, and S6, and strengthened the claim for an ACP pattern in S1 by identifying it in the Indian as well as the British and American samples. The Study 2 patterns in all of these statements were found in the China sample with one minor exception for S4.

Items S7 and S8, the statements involving migrants, exhibited AGP patterns in all four samples, with a minor exception in the Indian sample for S7. Notably, the AGP pattern occurred in the China sample, despite the question not only being in Chinese but also altered to refer to migration from one Chinese province to another. Implications regarding this pair of items for understanding when social identities are viewed as zero-sum are elaborated in Smithson et al. (2015).

## General discussion

In three studies, we have accumulated strong evidence that zero-sum-like statements are endorsed unequally when their elements are permuted, and these unequal endorsements exhibit systematic patterns that hold for adult samples from two Western and two non-Western cultures. In this section, we first characterize these patterns in terms of sets of preferences between pairs of alternative permutations of a zero-sum-like statement. We then briefly address the question of how strong these preferences are.

The patterns are listed in Table 9, with the differential endorsements listed as preferences for each pattern. For example, the ARF pattern includes the preferences Ab ≻ aB and Ba ≻ bA. The ACP pattern in Table 9 accords stronger belief to the effect of resource consumer A in the “if” part of the “if-then” statement than to the same effect of resource consumer B in the same position. This may reflect a belief that increases (decreases) in a resource to A drive their opposites in B to a greater extent than increases (decreases) to B drives A. For instance, participant data indicated beliefs that increases in immigration rates decrease available jobs but not the converse; and increased time spent at work decreases time spent in personal relationships but not the converse.

**Table 9:** **Patterns of differential zero-sum statement endorsement**.

ARF	*Ab* ≻ *aB*	*Ba* ≻ *bA*		
ACP	*Ab* ≻ *Ba*	*aB* ≻ *bA*		
AGP	*Ab* ≻ *aB*	*Ab* ≻ *Ba*	*bA* ≻ *aB*	*bA* ≻ *Ba*
Partial ARF + ACP	*Ab* ≻ *aB* ≻ *bA*	*Ab* ≻ *Ba*		
Partial ARF + Partial ACP	*Ab* ≻ *bA*	*aB* ≻ *bA*	*Ba* ≻ *bA*	

The AGP pattern, on the other hand, seems to reflect a belief that an increase in a resource to consumer A will decrease the resource for consumer B, and vice-versa, whereas declining fortunes for the rich will not necessarily benefit the poor. In the two national-identity items with the immigrants, increased identification with the country of origin is believed to decrease assimilation to the adopted country and vice versa, but decreased identification with the country of origin may not correlate with assimilation. One possible distinction between propositions yielding the AGP vs. the ACP pattern is that the AGP-yielding propositions may include a belief that consumer A is more likely than B to acquire more of the resource (e.g., the rich always will get richer, or the immigrant always will identify more strongly with the country of origin).

The ARF pattern in indicates a stronger endorsement of “if more then less” than “if less then more.” We did not observe a complete ARF pattern, but parts of it in connection with the ACP pattern. For instance, increased immigration decreases available jobs, whereas it is not as strongly believed that decreased immigration will increase available jobs. Thus, as suggested earlier, ARF patterns reflect beliefs about asymmetries in the excludability of resources, whereas the ACP pattern indicates beliefs about power differentials. The AGP pattern, on the other hand, combines these two components into a belief that there is a one-way flow of a resource to one consumer at the expense of the other.

The sizes of these effects are substantial, and this can be seen via the odds-ratio comparisons in Table 10. These are exponentiated mean coefficients for relevant samples, rescaled where necessary to use the bA pattern as the reference pattern. For example, the ACP entry is from the statements about time with friends vs. family, averaged over all four samples from Study 3. The Ab = 3.502 entry says that the odds of the Ab pattern being endorsed in a higher category is 3.502 times greater than the odds for bA, for any two-way split between the categories of the ordinal scale. These odds-ratios also echo the patterns of endorsement preferences for each corresponding pattern in Table 9.

**Table 10:** **Odds-ratio summary effect sizes**.

**Pattern**	**Odds-Ratio Summary Effect Sizes**	
**FRIENDS-FAMILY (FOUR SAMPLES FROM STUDY 3)**		
ACP	Ab = 3.502	bA = 1.000
	aB = 3.656	Ba = 1.574
**RICH-POOR (SEVEN SAMPLES FROM STUDIES 2 AND 3)**		
AGP	Ab = 0.913	bA = 1.000
	aB = 0.182	Ba = 0.132
**WORK-PERSONAL (FOUR SAMPLES (USA AND UK) FROM STUDIES 2 AND 3)**		
Partial ARF	Ab = 5.878	bA = 1.000
+ACP	aB = 3.375	Ba = 0.901
**BEST FRIEND (SIX SAMPLES (EXCLUDING CHINA) FROM STUDIES 2 AND 3)**		
Partial ARF	Ab = 1.819	bA = 1.000
+Partial ACP	aB = 1.799	Ba = 1.659

Our findings have implications for the study of zero-sum beliefs, regarding measurement, experimentation, and theory development. Starting with measurement, it is clear that permuting many zero-sum-like statements affects their endorsement in systematic ways. It is striking that a strict zero-sum endorsement pattern (treating the permutations as equally valid) was seldom observed in the three studies, even for statements where one could make a logical case that they are “objectively” zero-sum (e.g., tradeoffs between hours of sunlight and cloudiness). Moreover, the patterns of unequal endorsement were demonstrated to be robust and stable, not only across samples, but also between a 5- vs. 7-point response format. Nor is the list of such patterns likely to extend beyond the two that we anticipated and the third that we encountered in Study 2, as indicated by the fact that no additional patterns were revealed in Study 3. Finally, these effects are substantial and not ignorable, as indicated by the magnitudes of the odds-ratios reported in the ordinal logistic regression analyses.

How, then, may we measure and study zero-sum beliefs and the factors that influence them? Clearly the strength of belief in a zero-sum-like proposition cannot generally be captured entirely by ratings of one version of that statement. A prudent course is to present all four permutations of a zero-sum-like proposition and ascertain whether participants equally endorse them or whether they appear to be using a heuristic that renders them unequally believable, at least during the pilot-testing phase of a study. Unequal endorsements may reflect participants' beliefs about the power relations between the consumers, or stock and flow properties of the resources. It also should be noted that we have not dealt with cases where resources X and Y are free to be switched between consumers A and B. Such cases yield 8 possible permutations rather than just 4. They may arise in some types of trade relations (e.g., where countries A and B both may manipulate trade tariffs to influence each other's trade opportunities) and therefore are worthy of investigation.

Crucially, permutations of zero-sum-like statements may moderate the relationships between zero-sum thinking and individual differences measures of the kind investigated by Smithson and Shou (2016), or between zero-sum thinking and social psychological factors such as those reported in Smithson et al. (2015). It remains to be seen whether the patterns of unequal endorsement identified in our studies moderate the influence of such factors on the propensity to have zero-sum beliefs, although the Smithson and Shou (2016) work found no evidence of moderator effects of this kind. Nevertheless, given the current state of the art, measuring zero-sum beliefs necessarily involves two components: Strengths and configuration of the beliefs.

An implication of a framing interpretation is that these permutations may influence the extent to which people become “locked in” to a zero-sum orientation. This implication also is amenable to experimental tests. For example, Meegan's (2010) claim about people being more sensitive to others' gains than to others' losses is relevant here, because it is part of the ARF pattern. If members of group A are primed with the statement “The more X for group B, the less X for group A,” than may cause them to feel greater rivalry with group B than the statement “The less X for group B, the more X for group B.” Moreover, this effect may be moderated by the extent to which group A members believe groups A and B are in competition for resource X.

Finally, we return to the more general point in the introduction to this paper, noting that our findings essentially amount to a type of “order-effect” akin to, though distinct from, certain kinds found in attitude and decision research. One of the motivations behind this paper is to alert researchers to the possibility that permutation order-effects may exist in the measurement of other attitudes besides zero-sum beliefs. It seems plausible, for instance, that such effects are likely to be found in attitudinal statements that involve modus-ponens or conditionals (if A then B), association (A occurs with B), or causation (A causes B). In situations where researchers consider that either ordering (A-B or B-A) could be utilized for an item, it may be worthwhile to test for an order-effect.

## Author contributions

MS is the lead author of this paper. He originated the ideas behind the research, designed the studies, analyzed the data, interpreted the results, and took the lead on writing the paper. YS is the second author of this paper. She contributed to the study design, data management, data analysis and interpretation, and assisted with the write up.

### Conflict of interest statement

The authors declare that the research was conducted in the absence of any commercial or financial relationships that could be construed as a potential conflict of interest.
