# Supplementary material for: Asymmetries in Responses to Attitude Statements: The Example of “Zero-Sum” Beliefs
Source: Front Psychol. 2016 Jun 29;7:984. doi: 10.3389/fpsyg.2016.00984 (PMC4925710; doi:10.3389/fpsyg.2016.00984)
Supplement: Supplementary file 1 [file DataSheet1.docx]

Appendix A: Histograms for Study 2

S1: Work-Personal S2: Distance-Time

S3: Eat-Weigh S4: Best Friend

S5: Immigration-Jobs S6: Rich-Poor

S7: Food-Clothes S8: Cloudy-Sunny

Appendix B: Histograms for Study 3

S1: Work-Personal S2: Friends-Family

S3: Wealth S4: Best Friend

S5: Immigration-Jobs S6: Rich-Poor

S7: Al-Bayati S8: Al-Husseni
